# News and Miscellany

**Published:** 1883-07

**Authors:** 


### NEWS AND MISCELLANY.

Parasites in American Pork.—From an investigation into the parasites in the pork supply of Montreal, Dr. Osler concludes: 1. That the hogs slaughtered for our markets present parasites in numbers sufficient to necessitate a more thorough inspection than is at present carried out. 2. As regards trichina spiralis which was found in the proportion of 1 to 250, he is of opinion that, considering the extreme rarity of cases of trichinosis, and the difficulties attendant upon a systematic inspection, a compulsory microscopic examination of the flesh of every hog killed is not at present called for. 3. In the case of "measles" the liver should be carefully examined, and, if present in it, the flesh of the animal should receive the special attention of the inspector; if only in the liver the entire carcass need not not be confiscated. 4. Echinococcus cysts in the liver render that organ unfit for food, but in other parts, unless very numerous and disorganizing, they may be cut out, and the carcass remain marketable. 5. The public should be made aware of the possible dangers of eating, in any form, raw or partially cooked meat. The best safeguard against parasitic affections is not so much inspection of the flesh, unless, indeed, this is minutely carried out, as careful attention to culinary details. 6. To reduce the number of infected hogs greater attention should be paid to their hygienic surroundings, particularly in the matter of feeding. The danger is not during the period when the animals are penned and fed on grain, etc., but when they are allowed to roam at large and feed indiscriminately.

Presentation.—On the occasion of the closing of active operations with the stamping out of the Pictou Cattle Disease in Nova Scotia, Professor McEachran, the Veterinary Inspector in charge, was the recipient of a testimonial in the shape of a handsome Novia Scotia gold chain, accompanied by the following address, which was presented by Mr. George Caswell, on behalf of himself and fellow officers: "To William McEachran, Esq., M. D., Veterinary Inspector, Pictou. Dear Sir—On the closing of the operations in connection with the stamping out of the disease among the cattle of this country for this season, we, the undersigned officers employed under you in the work, beg to ask your acceptance of the accompanying chain as a small token of our esteem and regard for you personally, and also as an expression of our appreciation of the efficient manner in which you have conducted the measure for the stamping out of the disease. We would also express our thanks to you for the many acts of kindness and consideration shown to us personally by advice and assistance in the carrying out of our various duties, and would further testify of the uniform kindness and consideration with which you have dealt with the farmers and others with whom, in the discharge of what were often unpleasant duties, you have had to deal. Wishing you a safe journey homeward and prosperity for the future, we beg to remain, yours sincerely, George Caswell, Joseph Grant, James Grant, John McQueen, Alexander Traser, Lauchlin McInnis, Peter McInnis."

Professor McEachran replied in suitable terms, thanking them for their handsome expression of good will. He said that while the active operations were for a time suspended, he thought that it was the intention of the Minister of Agriculture to recommend active operation early in the spring, and hoped that another season would see this country completely rid of the plague which threatened ruin to so many.—*Veterinary Journal.*

Prof. Virchow's Health.—Our latest German exchanges announce a steady improvement in Prof. Virchow's health. He has been suffering for some time from an attack of acute nephritis, complicated by rheumatic neuralgia, and commencing with slight hemorrhage. He is now free from fever, and the amount of albumen in his urine is steadily declining.—*Denver Medical Times.*

RED WATER IN CATTLE.—A. B. Allen writes to the *National Live Stock Jonrnal* that red water is a very destructive disease to cattle in the Southern States, and thinks the best remedy is to keep the cattle free from injurious pastures and bad water. The English *Agricultural Gazette* says that if the disease is *taken in time* two ounces of spirits of turpentine, followed by two handfuls of salt, in buttermilk, have proved a sure cure. This is a simple remedy, and one that can do no harm even if it can do no good. Several English veterinary surgeons assert that sprinkling salt on pasture land has proved an effective remedy for red water.

ABNORMAL PERIODS OF GESTATION have been considered by naturalists and veterinarians who have given attention to these variations, rather as accidents than as an idiosyncrasy of individual habit or constitution. A considerable variation in the period may easily occur from physiological causes, as for instance the passage of the ovules through the fallopian tubes, which in the cow occupies four or five days. As fertilization cannot be completed until the ovule has been impregnated; this may be delayed as long as five days after the impregnation of the cow, by this movement of the ovule. Then for various reasons the birth may be delayed some time without injury, just as it may be prematurely hastened without injurious effect so long as the fœtus is mature. Thus a birth may occur 60 days before the normal period, it may be delayed just as long after it, and as 283 to 286 days is the average period, the shortest time may be 223 days, and the longest 343. The extremes, as given by various observers, are as follows: 241 to 301 days, average 283, by 1,062 observations made at the agricultural school at Saulsie by Blaine, Tessier, Grille and Fürstenburg; 210 to 353 days by Dieterich, average 286 days; 240 to 330 days by Baumeister and Reuff, average 285 days; 220 to 313 days by 764 observations by Earl Spencer, average 284 to 285 days. The American Journal of the Medical Sciences give as the result of observations for some years on 62 cows, 213 days as the shortest period, and 336 days as the longest.

Every observer, except Earl Spencer, states that the average gestation of bull calves is longer than that of females, and all the experience of breeders tends the same way, the difference being about six days. But there has also been a difference observed in regard to breeds. Wilhelm states that the Hungarian cow carries her calf ten days longer than the Dutch cow; the Swiss Simmenthal breed has an average period of 280½ days; the English cattle observed by Earl Spencer had an average of four days longer than this. These differences are supposed to be due to the fact that a very vigorous, robust and hardy animal will have a longer period than another which is pampered and forced, and whose breeding and keeping are more artificial and refined.

The subject is one of much interest to breeders and physiologists, more especially in regard to its development in such breeds as the Jerseys, Guernseys and Short-Horns, where close line breeding and unusually high culture are practiced, and in which any idiosyncratic differences might be expected, if they were possible at all.

Very similar variations have been noted in the mare, ewe, sow, and the smaller domestic animals, but no case is mentioned in which the variation has had more than an accidental significance that could be reasonably explained.—*Country Gentleman.*

PROFESSOR ROBERTSON, principal of the Veterinary College in London has in preparation a work on Equine Medicine, which will be ready in the autumn. William R. Jenkins will be the American publisher of it.

PERSONAL.—Henry W. Rowland, D.V.S., a graduate of Columbia Veterinary College, has been appointed United States Veterinary inspector to watch cattle traffic between New York and New Jersey.

HORSES FOR FOOD.—During the last six months 3,085 horses have been used for food in Berlin. The warm sausages sold in the streets at night are mostly of equine origin. When a prominent restaurant keeper failed some years ago the most conspicuous among his creditors was a horse butcher, which throws a side light on the "roast beef" that used to be served up to his guests. The poor are the chief buyers, but there are not a few gourmands who look upon horse flesh as a delicacy.—*Horseshoer and Hardware Journal.*

AIR-SPACE IN COW-SHEDS.—The regulations of the Metropolitan Board of Works as to cow-sheds fix an "air-space" of 800 cubic feet per cow, no height of shed above sixteen feet to be reckoned in measuring the space. At a recent meeting of the Dairymen's Association it was decided to memorialize the Board to reduce the "air-space" to 600 cubit feet. We trust that the Board will refuse to accede to this memorial. Eight hundred cubit feet per cow, represents the result of careful inquïry, confirmed by observation, of the minimum air-space which should be given to each cow in a shed, if the health of the animal is to be fully maintained.

CRUELTY TO ANIMALS.—Officer Evans, of the S. P. C. A., is doing good work in prevening ill treatment of cattle on the Hudson River steamers. It appears that the owners, in order to obtain large prices for their cows, are in the habit of muzzling the calves during the voyage to prevent them from drawing the milk, the result being that the udders become "bagged" and "caked."

THE Honorary Degree of L.L.D., of Cambridge, is to be conferred on M. Pasteur.

MORE VETERINARIANS NEEDED.—In an address before the Kentucky Medical Society, at its recent session, the practice of veterinary surgery was commended to young men. The speaker estimated that the yearly loss arising from the want of sound advice and treatment—the horses of the country being valued at nearly eight hundred million dollars—amounts to $15,000,000.

M. PASTEUR, the eminent microscopist and naturalist, to whose labors we owe most of our knowledge of the nature and behavior of the minute germs which produce various fatal disorders in animals, now receives from the French Government a pension of $5,000 yearly, to enable him to devote himself to continued studies.

A MISNOMER EXPLAINED.—The meaning of hy-dro-pho-bia is a dread of water, *not a disease* susceptible of connection by any absurd construction from a diagnosis of the *so-called* disease of canine madness, which exists only in the imagination. All persons, as well as animals, dread water or any drink when under the influence of excessive fear, as a morbid inactivity of the organs of the throat prevails which tends to quackle or choke, and this tendency in the canine race, after they have taken cold in wounds that produce spasms, is provervial. What has become of the mad dogs? In their absence let us calmly consider a few facts, and ask ourselves whether ignorance and superstition have not something to do with increasing their number and magnifying the dangers accompanying their malady. Dogs are sometimes afflicted with a distemper. When young they frequently have fits, running wild, with glaring eyes and frothing mouth; when old they have attacks of paralysis, and are reduced to a stupid, inactive condition, both of which have been called *rabies*. It is a common superstition, that should a dog go mad *after* biting a person, the latter will also fall a victim to rabies. Dogs suffering from wounds may take cold and inflammation setting in, the nerves become affected, spasms ensue, saliva is emitted, water is avoided, the whole appearance of the animal suggesting madness. The great disproportion of the sexes, occasioned by the destruction of the female dogs, has a capricious influence with various effects, including that of amativeness and jealousy, often making them irritable to strangers and to snap and bite them seemingly without cause, or at the least trifling annoyance even from friends. Persons taking cold in wounds have suffered in a precisely similar manner, with all the attendant manifestations of so-called rabies. The effect is the same as lockjaw, only that a wound from a rusty nail may, with inflammation from a cold, produce a stronger affection in the region of the throat. The end of many diseases which afflict humanity is attended with spasms, saliva, and other symptoms of hydrophobia. A few incidents will illustrate:

Some years ago a man in Dorchester, Mass., was bitten by a cat, another in Boston by a rat, and several by rabbits, the bites producing spasmodic symptoms in all the victims.

Mad horses and cows have been known, their disorder (frothing at the mouth, etc.,) being doubtless caused by a poisonous shrub eaten with hay. A father bitten by his child, from whose throat he attempted to remove diphtheritic formation, died from the wound. A blacksmith of Roxbury, Mass., sprained his ankle while attending to a horse; he took cold, inflammation ensued, then violent spasms, and paroxysms at intervals for a week preceding death. The newspapers of 1878 reported that last March, in New York, Mr. J. Russell was bitten in the hand by Thomas Kelly, while quarrelling with him, since then his finger, then his hand, then his arm were successively amputated. He finally died from the effects of the bite.

A farmer of Ohio died from the effects of a bite on the hand from an insane son. Some members of a family were poisoned from eating roast goose, which they imagined was bitten by a mad dog, and were undoubtedly under spastic delusion. Had these animals and persons been bitten by dogs, they would undoubtedly have been reported as victims of rabies.

A man was bitten on the hand by a dog in Kingston, N. Y., and after three years was seized with symptoms which were at once regarded as those accompanying hydrophobia by all the city physicians, who watched the case with much interest. Between the interval of spasms the patient was rational and quiet, partook of water and food at times, but *usually* the *sight* or *sound of water* threw him into violent spasms. The man recovered, and the physicians regarded it as *hysteria* with hydrophobia simulation, which they thought was merely mental.

A dog-trainer of New York, whose intelligent experience was of long standing, did not believe such a disease as canine madness existed. He was bitten, and while suffering from the wound his attendants called the malady hydrophobia; it was in reality delirium tremens. Watts, of Boston, who has probably the greatest experience with dogs in the New England States, never yet discovered evidence of this so-called disease. It is true that cases have been reported in the medical journals, but generally with a protest from eminent authorities. One instance is that of a woman, whose malady was hastily set down by the physician as hydrophobia. He was deceived by a chronic case of hysteric fits.

What gives something of a quietus to this obsolete supersition is the fact of such innumerable cases of persons having been bitten all over the world by dogs with no serious consequences which could be cited if space would admit, wherein rabies have been pronounced as absolutely existing in the animal and patient, without any foundation or fact, A single instance, for the present, gives the fancied ideal of a volume, which is from a person holding office in the Society for the Prevention of Cruelty to Children and Animals, who witnessed an extreme case, called rabies, of a dog in paroxysms, frothing and biting fences and everything in his way, and finally biting a lady severely, aside from which she experienced no ill effects, and lived thirty years thereafter. Last autumn a lawyer, of New York, was ferociously bitten by a large dog while entering the premises guarded by the faithful animal at night. He took care that he took no cold in the wound, and therefore no harm came of it. A lady of Cambridge, Mass., bitten by a black and tan pet last winter, took similar precaution with like result.

Hunters and sportsmen who have been bitten by their own and other dogs repeatedly, under various conditions and circumstances, some attacks being mild, others ferocious and of a mysterious nature, attest that no positive evidence has yet been produced to show that virus ever emanated from a canine's mouth.

The writer during his life has been surrounded by different species of dogs; his children and friends, as well as himself have often been bitten by them, sometimes severely; but by the exercise of every precaution against taking cold, no ill results have followed. If a wound be severe, first cauterize it, if possible; however this may be, the application of a poultice of flaxseed and slippery elm, saturated with laudanum, will remove all irritation. Many practitioners of Paris have for a long time doubted the existence of hydrophobia as a disease, and those of eminence, experience and deep research of Europe and America, regard it now as fallacious; while observation, experience, and an intelligent domiciliary view by others, who have studied the subject-matter, see no reason why this phantom should be a synonym of hydrophobia, with any more sense, than to substitute hallucination for reality.

J. G. Clapp.

[Our correspondent's statements are interesting, but they simply show that hydrophobia is a rare disease (which is well known,) and that many bites are harmless or only cause a non-specific tetanus.—Ed.]—*Medical Record.*

Veterinary Hospital.—The project in question has been handled in a practical way by the corporation of Harvard University, which has founded a school of veterinary medicine, and created, or has in process of preparation, a general establishment for the treatment of horses, cattle, sheep and dogs. This is to consist of a hospital in the city proper, and farm accommodations some four or five miles out, but still within the limits of the city. The hospital building, which is now in course of erection, is located at the junction of Village and Lucas Streets. It is to be constructed of brick with stone trimmings; will be 50x50 feet in area and three stories high, with a light, airy basement underneath. The entrance to the basement will be from Lucas Street, and down an incline. Here will be located the forge room, the stalls and other accommodations for cattle, sheep, etc., and for the boiler and other steam heating apparatus and machinery to run the elevator. On third floor will be a large quadrangular space, surrounded by the stalls for horses, etc. This is to be the lecture room. There are, also, one paded stall for violent cases, four large box stalls and six ordinary stalls. On the second floor, which is reached byan incline from the first, as well as by the elevator, there are four box stalls and five extra wide stalls, a large room for dogs, a pharmacy or drug room, and apartments for attendants. The third story will contain work-rooms, harness room, hay and grain loft, and a bedroom for the house surgeon. The elevator is to be of a capacity to take up the largest animal treated, and will be used in taking up the ailing horses, etc., brought for treatment. The building is to be heated with steam, and will have water on every floor. It will be thoroughly ventilated, and being constructed specially for the use to which it is dedicated, will contain every convenience and appliance which experience in older countries in similar lines of treatment have suggested. The stalls are of hard pine and iron, and will be constructed after the most approved models. There will be accommodations for ten horses at a time, and space for a few cows and and other animals; the room for dogs is to be properly fitted up with all the modern improvements for the cure of the sick bow-wows. The patients in the hospital will be under the professional charge of Dr. Charles P. Lyman, fellow of the Royal College of Veterinary Surgeons of London, England, and professor of veterinary at Harvard University. In addition to the hospital just described, the hospital management will have at its disposal a large farm, with comfortable accommodations and stone buildings at West Roxbury, near the Forest Hill station of the Boston and Providence railroad, where cattle can be received and cared for, and where horses not required for present use, or that may be suffering from lameness, or illness requiring long treatment of rest, can be sent, and where they will be sure of receiving proper care and treatment. Here they can also have the benefit of grass paddock or pasture in the summer season, and of a warm, comfortable straw yard in the winter.

Illinois State Veterinary Convention.—A large number of veterinary surgeons assembled in Chicago, May 22d, with the object of forming the "Illinois State Veterinary Medical Association." The Committee on Constitution and By-laws reported a preamble, which was adopted as a whole. It called attention to the fact, that the practice of veterinary medicine and surgery stands in close alliance with the practice of human medicine in point of importance and necessity; that the general public recognize this, and are more inclined to accord to veterinarians their proper position, socially and professionally; that the association of professional brethren exerts a powerful influence in promoting friendship and unity, breaking down the barriers of formality and personal jealousies, and at the same time providing ways and means for the discussion of topics of mutual interest, and in consideration of these and other reasons, resolved to form a mutual benefit and protective association, to be known as "The Illinois State Veterinary Medical Association." The constitution was then read, and the Convention elected the following officers:

President—A. H. Baker, of Chicago.
1st Vice-President—Wm. Sheppard, of Ottawa.
2d Vice-President—I. J. Miles, of Chicago.
3d Vice-President—J. D. Tuthill, of Charlston.
Recording Secretary—Joseph Hughes, of Chicago.
Corresponding Secretary—John F. Ryan, of Chicago.
Treasurer—W. L. Williams, of Bloomington.
Board of Censors—Wm. Sheppard, of Ottawa; R. J. Withers, of Chicago; N. H. Paaren, of Chicago.

The By-laws were adopted, and, together with the Constitution, signed by all the members present. The Convention was then declared at an end, and the "Illinois State Veterinary Medical Association" was called to order by President Baker. Communications were then read from Prof. Bates of Columbia College, Prof. McEachran of Montreal, Prof. Liautard of New York, Prof. Smith of Toronto, Prof. Lyman of Harvard College, Prof. Jennings of Detroit, D. P. Frame of Burlington, and several others, regretting their inability to be present, and extending their best wishes for the success of the movement, and congratulations on the formation of the Society.

The Chairman then nominated Drs. Sheppard, Hale and Withers a committee to draft a bill to be presented to the Legislature for their action.

Dr. William Sheppard, of Ottawa, read a paper entitled "Lameness in Horses," receiving a vote of thanks.

Dr. Withers then highly complimented the manager and editor of the *U. S. Veterinary Journal* on the successful issue to which, through its medium, the Convention had been brought.

The Degree of Doctor of Laws.—The members of the veterinary profession will be pleased to learn that, by resolution of the Senatus Academicus of the University of Glasgow, his native city, the degree of LL.D. was conferred on Mr. George Fleming, Army Veterinary Inspector, at the graduation there on April 27th, in recognition of the services he has rendered to medical and veterinary science, and particularly to comparative pathology and sanitary science. We believe this is the first time that a university has acknowledged the claims of veterinary science to the highest honor the Senate can confer.—*Veterinary Journal.*

Presentation.—Mr. George Fleming, President of the Royal College of Veterinary Surgeons, has lately been the recipient of a testimonial, subscribed for by members in every part of the world. In reply to the address of Mr. Greaves on behalf of the profession, Mr. Fleming said that the object of his life had been to promote the instruction, elevation and welfare of the profession. From his early days he had been deeply impressed with the necessity of promoting veterinary science in England and all English speaking countries, and procuring for the profession a social station hitherto denied it. He felt now that they recognized that he had done his duty in that direction. The subscriptions amounted to over £450, and was spent in having two portraits of Mr. Fleming painted, one for himself and the other for the Royal College of Veterinary Surgeons, and in the purchase of the presents, which consisted of two pieces of silver plate and a diamond ring.

GLANDERS has appeared among the horses of Whiteside and Perry counties in Illinois, and two persons, father and son, have died, having become inoculated with the poison while caring for their sick horses. Strict measures have been taken by the authorities to prevent a spread of the disease, a number of the animals having been shot and others quarantined, and it is believed that the disease is now entirely under control.—*Weekly Medical Review.*

THE TREATMENT OF IMPORTED CATTLE.—The Secretary of the Treasury has issued a circular to customs officers promulgating regulations governing the treatment and quarantine of imported cattle as follows: All cattle arriving in the United States from Europe, Asia, Africa, Austrialia or New Zealand shall be subjected to a quarantine of ninety days, counting from the date of shipment. It shall be the duty of the veterinary inspector at each port to see that the cattle imported shall be securely guarded against the risk of transmitting or receiving contagion until they shall have entered the quarantine grounds, and all imported cattle shall be under his control from the time of landing until they reach the quarantine grounds. He shall also be superintendent of the quarantine, and shall have charge of the grounds, buildings, yards, and all property thereto belonging.

DR. L. ROTH, of Kissengen, reports a violent outbreak of diphtheria among a barnyard of fowls, which he attributes to infection from children, some of the poison being mixed with the sweepings of the room and thrown into the yard. The *Med. Press*, March 21, 1883, says: "Such an observation as this is very interesting, and seems to bear out some of the statements of Herr M. Wolff in a paper 'On a Widespread Brute Mycosis.' In it he drew attention to the infectious diseases of domestic animals, which clinically and anatomically run a course exactly similar to those of human beings. He mentioned the fact that anthrax was met with in fowls, geese and ducks, and caused the same phenomena as when its habitat was the mammalia. He also mentioned another devastating mycosis prevalent amongst domestic birds that bore a complete analogy to diphtheria (*vide* Dr. Roth's observation). Yellow and white-yellow membranes were developed upon the most diverse mucous membranes, having all the characteristics of human diphtheria, so that it could not be removed without causing bleeding. A third disease that had its analogue in man was ulcerative endocarditis, that runs its course with the same valvular changes and multiple emboli in the various organs as in man."—*Weekly Medical Review.*

VACCINATION,—M. Peters, a member of the Academy of Paris, has declared before that learned body that animal vaccination by the Pasteur method is an uncertain, useless and dangerous proceeding. There is a veterinary department at the Berlin Hygienic Exhibition which shows in a thorough manner various methods of treating animals when vicious or in pain. There is a complete line of horse-shoes, from the most ancient days known to the present time, and an exhibition of calculi found in the large intestines of horses which would certainly excite much interest in the minds of our skilful surgeons. The largest is the size of a man's head.

COPPERHEAD VENOM.—Dr. I. Ott (*Virginia Medical Monthly*, February, 1883), comes to the following conclusions:

"1. The venom of the copperhead is weaker in toxic activity than that of the rattlesnake.

"2. The heart, with both kinds of venom, becomes greatly prostrated, and in rapid deaths is their main cause.

"3. The venom of either snake does not affect the sensory nerves.

"4. The sensory nerves are affected by both venoms.

"5. The muscular excitability continues to be little affected at the time of death by the poison of the copperhead.

"6. The two venoms greatly resemble each other in physiological activity.

"7. The cardiac force, rhythm, and frequency are lowered by both venoms.

"8. The arterial tension is greatly lowered by both venoms.

"9. The blood, after copperhead poisoning, shows uo microscopic changes of its globules, and no difference in its spectrum."

ARE THERE INSANE ELEPHANTS?—To the zoölogist this question seems absurd, but the existence of insanity among elephants has been denied by the dilettante alienists of the London *Lancet*, who are often remarkable for their misinformation on human psychiatry and histology, and can scarcely be expected to have a speaking acquaintance with certain primary facts in comparative psychiatry. The insane elephant is so common in countries where the elephant exists as to be designated by a popular name. He is called a "rogue." In the case reported by Dr. Todd, of St. Louis, in this Journal, January, 1881, p. 61, the autopsy on an elephant who suddenly became violent and destructive, having been always well behaved, revealed marked meningeal and encephalitic congestion. What were the changes found in the case of the elephant Pilot, recently slaughtered by Barnum because of insanity, are not yet known, but he was decided insane. The *Lancet* seems to need a good instructor in histology and psychiatry very much.

TRICHINÆ.—Mr. Frank S. Billings, veterinary surgeon, contributes two valuable articles to the New York *Medical Journal* upon "Trichiniasis in its relations to the Public Health and National Economy." His method is to take a stump of the pillars of the diaphragm and examine their sections. Of the 8,773 hogs examined 345 were found infected, that is one in every twenty-five. This result, says Mr. Billings, does not certainly support the words of the State Department, that there are "less trichinæ in American pork than that of any other country. Comments are made upon the manner in which some investigations have been made. In one case an objective capable of magnifying two hundred diameters was used, and the pieces examined were separated into shreds. Mr. Billings very pertinently remarks that as the length of the female trichina is one-eight of an inch, he should expect, when he did happen to find one, to find a boa-constrictor. In the second place, it is well known that the parasite is most easily recognized by using crushed specimens. In conclusion, he says:

"Some unknown living thing lodges trichinæ before they enter the porcine organism. The questions are: What is it? Where is it? What are its modes of life? These things discovered, and they must be, an end can be put to porcine trichiniasis, and hence to human. Bluffing the attempt to write down an existing fact will only end in ridicule to this government; research, untiring and unceasing, is the only 'cure'; cost what it will, it will pay far better than government lies. American hogs are much more trichinous than European."—*San Francisco Western Lancet.*

Cerebral Congestion.—A grey c. b. gelding, 11 years old, while under treatment for a contused wound of elbow, caused by a kick, was found with his head over the box wall, sweating profusely with 80 respirations per minute. heard at a considerable distance, anxious expression and struggling for breath. While being observed he sank down with his head resting on his knees, but standing upright on his hind limbs—an unusual position for a horse. After a few moments he dropped into a heap in a corner and remained quiet, except when efforts were made to apply remedies, which, from the danger involved, were finally abandoned, dying in the evening of the day following.

Autopsy revealed broken down kidneys, structure, melanotic tumor over right external illiac vein, rupture of spleen closed by a plug of fibrin, lungs congested, partly gangrenous, and their vessels filled with thrombi, heart cavities and vessels arising from them for some distance filled with clots, brain congested, membranes ecchymosed and depressed fracture of frontal bone. Cause of death, thrombosis.

The case is of interest from the fact that the loud breathing caused by pressure of throat against the wall was a misleading sympton, in the remarkable attitude and in the proof that a rent spleen is not immediately fatal.

Cerebral Tumor.—An eight-year-old bay gelding was observed one morning to be off feed and dull, with staggering gait. slow and full pulse, injected mucous membranes, depressed head, tympanic abdomen, and incontinence of urine. These symptoms improved under treatment and the patient continued to improve until the evening of the third day, when the following symptoms developed themselves: Head enormously enlarged, eyes closed, nostrils obliterated by effusion, temperature 105°, slow and stertorous breathing, cold sweat, continuous spasmodic movement of lower jaw and near hind extremity and swaying to and fro of body. The movement of jaw and leg did not cease until death, which took place on the fourth day.

*Autopsy.*—Abdomen healthy; cerebrum covered with blood; veins congested and enlarged; arteries empty, but no clots; "mother of pearl" like tumor in lateral ventricle composed of cholesterine and fatty matter.

The case is of interest from the remission of the symptoms and the continous movement of hind limb and lower jaw.—*Quarterly Journal of Veterinary Science in India.*

There was recently exhibited at West Philadelphia a very simple method, and an improvement upon the Rarry system of training horses, and the manner in which some of the wildest horses were subdued was astonishing. The first trial was that of a kicking or "balking" mare, which her owners said had allowed no rider on her back for a period of at least five years. She became tame in about as many minutes and allowed herself to be ridden about without a sign of her former wildness. The means by which the result was accomplished was a piece of light rope, which was passed around the front jaw of the mare just above the upper teeth, crossed in her mouth, thence secured back of her neck. It was claimed that no horse will kick or jump when thus secured, and that a horse after receiving the treatment a few times will abandon his viscious ways forever. A very simple method was shown be which a kicking horse could be shod. It consisted in connecting the animal's head and tail by means of a rope fastened to the tail and then to the bit, and then drawn tightly enough to incline his head to one side. This, it is claimed, makes it absolutely impossible for a horse to kick on the side of the rope. At the same exhibition a horse, which for many years had to be bound to the ground to be shod, suffered the blacksmith to operate on him without attempting to kick while secured in the manner described.—*Chicago Tribune.*

